# Heterozygosity–fitness correlation at the major histocompatibility complex despite low variation in Alpine ibex (*Capra ibex*)

**DOI:** 10.1111/eva.12575

**Published:** 2017-12-04

**Authors:** Alice Brambilla, Lukas Keller, Bruno Bassano, Christine Grossen

**Affiliations:** ^1^ Department of Evolutionary Biology and Environmental Studies University of Zurich Zurich (ZH) Switzerland; ^2^ Alpine Wildlife Research Centre Gran Paradiso National Park Noasca (TO) Italy

**Keywords:** Alpine ibex, bottleneck, heterozygosity–fitness correlation, infectious keratoconjunctivitis, MHC

## Abstract

Crucial for the long‐term survival of wild populations is their ability to fight diseases. Disease outbreaks can lead to severe population size reductions, which makes endangered and reintroduced species especially vulnerable. In vertebrates, the major histocompatibility complex (MHC) plays an important role in determining the immune response. Species that went through severe bottlenecks often show very low levels of genetic diversity at the MHC. Due to the known link between the MHC and immune response, such species are expected to be at particular risk in case of disease outbreaks. However, so far, only few studies have shown that low MHC diversity is correlated with increased disease susceptibility in species after severe bottlenecks. We investigated genetic variation at the MHC and its correlations with disease resistance and other fitness‐related traits in Alpine ibex (*Capra ibex*), a wild goat species that underwent a strong bottleneck in the last century and that is known to have extremely low genetic variability, both genome‐wide and at the MHC. We studied MHC variation in male ibex of Gran Paradiso National Park, the population used as a source for all postbottleneck reintroductions. We found that individual MHC heterozygosity (based on six microsatellites) was not correlated with genome‐wide neutral heterozygosity. MHC heterozygosity, but not genome‐wide heterozygosity, was positively correlated with resistance to infectious keratoconjunctivitis and with body mass. Our results show that genetic variation at the MHC plays an important role in disease resistance and, hence, should be taken into account for successfully managing species conservation.

## 1 INTRODUCTION

The ability to fight disease and to adapt to novel pathogens is crucial for population survival but knowledge about the exact mechanisms driving disease resistance in wild species is still limited (Acevedo‐Whitehouse & Cunningham, 2006; Buitkamp et al., 1996; Radwan et al., 2010; Spielman et al., 2004). Because of their vulnerability to disease outbreaks, such knowledge is especially needed for endangered and reintroduced species.

A gene region known for its role in disease susceptibility is the major histocompatibility complex (MHC), a large gene complex that encodes proteins involved in immunity. The adaptive immune response in vertebrates is mainly driven by the MHC, and most of the loci identified within this region are expressed and encode for MHC molecules that bind immunogenic peptides inside cells and present them to lymphocytes for activation of the immune response (Janeway et al., 2001). The MHC is known to be one of the most variable regions of the entire vertebrate genome (Janeway et al., 2001). Its high variability is due to the large number of genes, several of which are furthermore highly polymorphic and this greatly increases the number of pathogens recognized by the immune system. This high diversity leads to high degrees of heterozygosity at the MHC. Moreover, due to the high polymorphism, individuals in a population will differ in the combinations of MHC molecules they express increasing the chance that to any given pathogen there will be a resistant individual.

High levels of genetic variation at the MHC are maintained through balancing selection (Hedrick & Thomson, 1983; Garrigan & Hedrick, 2003; Aguilar et al., 2004; Hughes & Nei, 1989, 1998; Hughes, Hughes, Howell, & Nei, 1994). The three mechanisms proposed to explain the advantage of MHC variability are as follows: a) overdominance or heterozygote advantage, b) rare allele advantage, and c) fluctuating selection. Theory predicts that any of the three mechanisms or any combination of them could maintain MHC diversity (Spurgin & Richardson, 2010). In addition, van Oosterhout (2009) proposed a model in which recessive deleterious mutations accumulate nearby MHC genes and thereby reduce the allelic turnover. Although it is not easy to identify the exact mechanism by which higher variation at the MHC is maintained (Spurgin & Richardson, 2010), it was shown that low MHC diversity can lead to higher susceptibility to disease and lower fitness (Bernatchez & Landry, 2003). Some studies investigated the correlations between MHC heterozygosity (at single or multiple loci) and fitness or disease resistance but most of them found associations with specific alleles. Evidence mainly comes from experimental studies as, for instance, on frogs *(Silurana tropicalis*, Savage & Zamudio, 2011) and on the endangered chinook salmon (*Oncorhynchus tshawytscha*, Arkush et al., 2002) but correlations between MHC variation and fitness or disease susceptibility were also found in wild populations (Aguilar et al., 2016; Bateson et al., 2016; Lei, Zhou, Fang, Lin, & Chen, 2016; Osborne, Pearson, Chilvers, Kennedy, & Gemmell, 2015; Paterson, Wilson, & Pemberton, 1998).

Many species, which went through severe bottlenecks, show very low levels of genetic diversity at the MHC, for example, mountain goats, *Oreamnos Americanus* (Mainguy, Worley, Côté, & Coltman, 2007) and Galàpagos penguin, *Spheniscus mendiculus* (Bollmer, Vargas, & Parker, 2007). Due to the observed link between the MHC and disease susceptibility, such species are expected to be especially vulnerable to disease outbreaks. Hence, there is a special interest in the interaction between MHC variation and disease resistance in conservation efforts. However, so far only few studies have shown that low MHC diversity is correlated with increased disease susceptibility in wild species after severe bottlenecks (Bateson et al., 2016; Osborne et al., 2015).

A wild species, which recently suffered local severe reductions as a consequence of epidemic diseases, is the Alpine ibex (*Capra ibex*), a wild mountain ungulate inhabiting the Alpine arc. Due to the recent demographic history, Alpine ibex represent an ideal model to investigate potential effects of MHC variation on disease susceptibility and more generally on fitness in an inbred, bottlenecked species. Due to overhunting, this species underwent a severe bottleneck during the XIXth century. Only about 100 individuals survived in the region now a days included in the Gran Paradiso National Park (GPNP). Alpine ibex survived thanks to the creation of a Royal hunting reserve followed by the establishment of the Gran Paradiso National Park. Widespread reintroductions have since re‐established Alpine ibex populations across the Alpine arc (ca. 50,000 individuals in 2013, GPNP, unpublished data). Nevertheless, genetic consequences of the bottleneck history are still visible in all ibex subpopulations (Biebach & Keller, 2009; Grossen, Biebach, Angelone‐Alasaad, Keller, & Croll, 2017). Observed heterozygosity at microsatellites of Alpine ibex is one of the lowest registered in wildlife (*H*_o_ = 0.4), standardized number of alleles per marker is low (Na = 2.44) (Biebach & Keller, 2009), and low diversity was also confirmed in another study based on genome‐wide SNP markers (Grossen et al., 2017). In addition, a recent study in the Gran Paradiso population found evidence for inbreeding depression in this species: Males with lower genome‐wide heterozygosity (evidence for higher inbreeding) showed lower body mass, shorter horns, and higher parasite burden compared with the average of the male population (Brambilla, Biebach, Bassano, Bogliani, & von Hardenberg, 2015).

Despite the general recovery of the species after the bottleneck, concern about disease susceptibility of Alpine ibex emerged during the last years. Some recently reintroduced colonies became extinct or almost extinct as a consequence of epidemic diseases (e.g., sarcoptic mange in the Eastern Alps, Carmignola, Stefani, & Gerstgrasser, 2006). Other populations showed strong seroprevalence of pathogens once common only in domestic ruminants (e.g., infection from *Brucella melitensis* in the Bargy massif (France), Mick et al., 2014) and also the well‐established population of Vanoise (France) suffered a severe numerical reduction due to a pneumonia outbreak (Garnier, Gaillard, Gauthier, & Besnard, 2016).

A disease that has recurrently affected Alpine ibex populations and led to population reductions is the infectious keratoconjunctivitis (Giacometti, Janovsky, Belloy, & Frey, 2002). In 2005–2008, the source population of Gran Paradiso National Park experienced a severe outbreak of infectious keratoconjunctivitis (IKC). This highly contagious ocular infection is caused by different strains of *Mycoplasma conjunctivae* (Belloy et al., 2003; Gelormini et al., 2017; Giacometti et al., 2002). The disease is characterized by an inflammation of the cornea and the conjunctiva with different stages of ocular lesions; in the most acute stage, the cornea can be perforated leading to blindness (Mayer, Degiorgis, Meier, Nicolet, & Giacometti, 1997). In wildlife, it can indirectly cause high mortality (up to 30%, Giacometti et al., 2002). If blindness‐related death does not occur, recovery from the disease is frequent. Despite lower than in symptomatic animals, *Mycoplasma* load is also present in healthy carriers and the pathogenesis of infectious keratoconjunctivitis is thought to be influenced, among other factors, by host predispositions (Mavrot et al., 2012). It remains unknown whether genetic factors may explain differences in susceptibility to the disease, levels of infections, and recovering probability.

A recent study on Alpine ibex highlighted that genetic variation at the MHC region is low (Grossen, Keller, Biebach, & Croll, 2014). Moreover, the observed variation was generated by recent introgression from domestic goat (Grossen et al., 2014). Without the introgression, Alpine ibex would be monomorphic at the generally highly polymorphic DRB exon II locus. This introgression may therefore have been adaptive (Grossen et al., 2017). However, so far no study has looked at potential fitness consequences for Alpine ibex linked with this locus. As Gran Paradiso is the source of all existing Alpine ibex populations, higher genetic diversity was expected in that population. Slightly higher genome‐averaged variation in Gran Paradiso than the reintroduced populations was indeed found using microsatellites (Biebach & Keller, 2009) and SNP data (Grossen et al., 2017) but Grossen et al. (2014) did not find higher variation at two MHC‐linked markers. The low diversity at the MHC observed in Alpine ibex could be one of the factors explaining disease susceptibility in this species. However, although it might be crucial for the conservation of this and of other bottlenecked species, the previous studies did not have access to individual‐based fitness data including disease susceptibility. Hence, the effect of variation at the MHC region on fitness and disease resistance in Alpine ibex is currently unknown.

The aims of this study, conducted in the source of all existing Alpine ibex populations, were 1) to estimate genetic variation at the MHC region using more markers and a larger sample size than previous studies in the Gran Paradiso population and 2) to investigate whether variation at the MHC region (measured as multilocus heterozygosity or heterozygosity at single loci) is correlated with fitness‐related traits and particularly with resistance to infectious keratoconjunctivitis.

## 2 METHODS

### 2.1 Study site, populations, and sampling

The study was conducted in the source population of all existing Alpine ibex populations, the Gran Paradiso National Park (North‐Western Italian Alps). This population is one of the few ibex populations with individually marked animals and the only one with reliable long‐term fitness data of individuals with known genotypes. Male Alpine ibex were captured, sampled, and marked in the framework of a long‐term monitoring project started in 1999. Details on captures and marking protocols are described in Brivio, Grignolio, Sica, Cerise, and Bassano (2015). At one study site (Levionaz), nearly the entire male population was captured, individually marked, and observed daily allowing also the collection of data on fitness‐related traits for the whole life of marked individuals. The number of females marked and for which fitness data are available was not enough to add them to the analysis. For this study, *N* = 247 samples were collected for genetic analysis in Gran Paradiso National Park. Of these samples, *N* = 147 were collected in the Levionaz study site, together with data on fitness‐related traits (see next section for details), and *N* = 100 samples were collected in other areas of Gran Paradiso National Park. For details on sample collection and storage, see Brambilla et al. (2015). Genetic diversity measures were calculated using all 247 samples, while heterozygosity–fitness correlations were performed only on the 147 samples of the Levionaz study site.

### 2.2 Fitness‐related traits data collection

For the 147 individuals of the Levionaz study site, which were born between 1985 and 2009, we collected data on two fitness‐related traits: body mass and annual horn growth. To estimate body mass, repeated weight measurements were taken during summer (from May to September) using an electronic platform scale baited with salt (Bassano, von Hardenberg, Pelletier, & Gobbi, 2003). The yearly body mass used in this study was defined as weight estimated at the 1st of August (von Hardenberg, 2005).

Ibex annual horn growth can be measured using visible horn annuli. During captures or if an animal was found dead, a caliper was used to measure the annual horn growth along a central line on the external side of the horns. For animals still alive, annual horn growth after capture was based on remote measurements using pictures following Bergeron (2007) or Brambilla and Canedoli (2014). Both horns were measured and the mean of the two was taken as horn growth per year. To avoid issues of variance heterogeneity, only horn measurements taken between the ages of 3 and 12 years were used for further analysis.

During an infective keratoconjunctivitis epidemic, which occurred in the Gran Paradiso population between 2005 and 2008, individuals of the Levionaz study site were monitored for symptoms of eye infections. The health status of marked males was assessed during captures or through detailed visual inspection of pictures of the eyes repeatedly taken during each year of the disease outbreak. In each year of the disease outbreak, male ibex were assigned to one of the following categories: 1 (sick/infected) or 0 (not infected, or asymptomatic host/carriers). Information on survival and recovery of infected animals was also collected. Laboratory investigations on two samples (isolation and PCR) identified the isolate as *Mycoplasma conjunctivae* (R. Orusa, personal communication).

### 2.3 Microsatellite genotyping

We genotyped all 247 GPNP individuals at 37 selected polymorphic putatively neutral microsatellites (Biebach & Keller, 2009; Brambilla et al., 2015). We also genotyped the same individuals at six MHC‐linked microsatellites: OLADRB1, OLADRB2, OMHC1, Bf94.1, BM1258, and BM1818 (Table 1). The microsatellites OLADRB1 and OLADRB2 are located within the MHC class II region. OLADRB1 has previously been shown to be diagnostic in Alpine ibex for the sequence at the second exon of the locus MHC DRB (Alasaad et al., 2012; Grossen et al., 2014). OLADRB1 is located in the intron directly after the second exon of the DRB locus (Schwaiger, Buitkamp, Weyers, & Epplen, 1993). Allele 184 was completely associated with the introgressed DRB exon 2 allele (Alasaad et al., 2012; Grossen et al., 2014). OLADRB2 is located about 100‐160 kb away from OLADRB1 (distance based on homologous sequences in cattle and sheep). Allele 277 was completely associated with allele 184 of microsatellite OLADRB1 (except for two of 707 individuals, Grossen et al., 2014). Microsatellite OMHC1 is located within the MHC class I region (Groth & Wetherall, 1994). Bf94.1 is located adjacent to the complement factor B gene, which is located within the MHC class III region (Groth & Wetherall, 1995). BM1258 and BM1818 (Maddox et al., 2001) are flanking the MHC region, about 9.5 Mb and 6 Mb away (distances from domestic goat). Protocols for DNA extraction and genotyping are described in Brambilla et al. (2015) and Biebach and Keller (2009).

**Table 1:** Description of the fixed part of the model set built to test heterozygosity–fitness correlations for body mass and horn growth

Model description	Model notation of the fixed effect	AICc Body mass model	AICc Horn growth model
Age^2^	Trait_*ij*_ = **β**_***0***_ * + * **β**_***1***_ *·*Age_*ij*_ * + * **β**_***2***_ *·*Age^2^_*ij*_ * + *ε_*ij*_	**2692.022**	**2804.311**
Age^2^, MLH neutral	Trait_*ij*_ = **β**_***0***_ * + * **β**_***1***_ *·*Age_*ij*_ * + * **β**_***2***_ *·*Age^2^_*ij*_ * + * **β**_***3***_ *·*MLH_n_*ij*_ * + *ε_*ij*_	2693.307	**2800.748**
Age^2^, MLH neutral, age interaction	Trait_*ij*_ = **β**_***0***_ * + * **β**_***1***_ *·*Age_*ij*_ * + * **β**_***2***_ *·*Age^2^_*ij*_ * + * **β**_***3***_ *·*MLH_n_*ij*_ * + * **β**_***4***_ *·*(MLH_n·Age)_*ij*_ * + *ε_*ij*_	2694.172	**2802.525**
Age^2^, MLH MHC	Trait_*ij*_ = **β**_***0***_ * + * **β**_***1***_ *·*Age_i*j*_ * + * **β**_***2***_ *·*Age^2^_*ij*_ * + * **β**_***3***_ *·*MLH_MHC_*ij*_ * + *ε_*ij*_	**2688.367**	2805.404
Age^2^, MLH MHC, age interaction	Trait_*ij*_ = **β**_***0***_ * + * **β**_***1***_ *·*Age_*ij*_ * + * **β**_***2***_ *·*Age^2^_*ij*_ * + * **β**_***3***_ *·*MLH_MHC_*ij*_ * + * **β**_***4***_ *·*(MLH_MHC·Age)_*ij*_ * + *ε_*ij*_	**2689.248**	2806.794
Age^2^, both MLH	Trait_*ij*_ = **β**_***0***_ * + * **β**_***1***_ *·*Age_*ij*_ * + * **β**_***2***_ *·*Age^2^_*ij*_ * + * **β**_***3***_ *·*MLH_n_*ij*_ * + * **β**_***4***_ *·*MLH_MHC_*ij*_ * + *ε_*ij*_	3343.984	**3104.273**
Age^2^, both MLH, age interaction	Trait_*ij*_ = **β**_***0***_ * + * **β**_***1***_ *·*Age_*ij*_ * + * **β**_***2***_ *·*Age^2^_*ij*_ * + * **β**_***3***_ *·*MLH_n_*ij*_ * + * **β**_***4***_ *·*MLH_MHC_*ij*_ * + * **β**_***5***_ *·*(MLH_n·Age)_*ij*_ * + * **β**_***6***_ *·*(MLH_MHC·Age)_*ij*_ * + *ε_*ij*_	**2691.284**	**2804.345**

All models also included individual id and year as random effects. Models with AICc values reported in boldface (ΔAICc < 4 compared to the best model) were used for model averaging.

MLH, multilocus heterozygosity.

## 3 DATA ANALYSIS

### 3.1 Genetic diversity and heterozygosity in the Gran Paradiso population

Microsatellites were divided into two sets: one set of 37 putatively neutral and genome‐wide distributed markers (selected by Brambilla et al., 2015) and a set of six MHC‐linked microsatellites. Diversity measures were estimated using data from 247 individuals sampled in the Gran Paradiso population using Genodive (Meirmans & Van Tienderen, 2004). These measures included number of alleles, effective number of alleles (calculated as the number of equally frequent alleles it would take to achieve a given level of gene diversity), and heterozygosity. Tests for Hardy–Weinberg equilibrium and linkage disequilibrium were performed in Genepop (Raymond & Rousset, 1995).

Standardized multilocus heterozygosity was calculated for both marker sets: 1) multilocus heterozygosity at neutral microsatellites (*from here on*: neutral heterozygosity) and 2) multilocus heterozygosity at MHC microsatellites (*from here on*: MHC heterozygosity). Standardized multilocus heterozygosity was calculated for each individual as the ratio of its heterozygosity to the mean heterozygosity in the population of the loci at which the individual was genotyped (Coltman, Pilkington, Smith, & Pemberton, 1999). This measure avoids bias due to possible differences in loci typed between individuals.

To detect possible directional changes in heterozygosity over time in the Gran Paradiso population, we compared allele frequencies of MHC‐linked microsatellites in three subsets of the data: Alpine ibex individuals sampled before 2001 (*N* = 87), between 2002 and 2004 (before the disease outbreak, *N* = 57), and between 2009 and 2011 (after the end of the disease outbreak, *N* = 37).

### 3.2 Heterozygosity–fitness correlations

Heterozygosity–fitness correlations were performed only on the 147 male individuals of the Levionaz study site for which data on fitness‐related traits were available.

We were specifically interested in potential heterozygosity–fitness correlations with MHC markers. Such a correlation is, however, only meaningful if patterns observed at the MHC are independent from neutral diversity. Therefore, to test for independence of the two sets of markers, the correlation between neutral heterozygosity and MHC heterozygosity was calculated. Similarly, to test whether individuals with similar MHC heterozygosity were more closely related than individuals with different MHC heterozygosity, we performed a principle component analysis (PCA). The PCA was based on a matrix of covariances of individual allele frequencies (0, 0.5, 1) at the neutral microsatellites and performed in Genodive (Meirmans & Van Tienderen, 2004). MHC heterozygosity of each individual was illustrated using differing point size (larger for higher MHC heterozygosity). This allowed visual inspection of potential clustering of individuals with similar MHC heterozygosity.

The power to detect heterozygosity–fitness correlations is higher if identity disequilibrium is nonzero (Miller & Coltman, 2014). To assess identity disequilibrium, g_2_, a measure of the covariance in heterozygosity, was estimated using the software RMES (Robust Multi‐locus Estimates of Selfing, David, Pujol, Viard, Castella, & Goudet, 2007). The analysis was performed on the Levionaz individuals with the six MHC‐linked microsatellites and 1,000 iterations.

To test for correlations between MHC heterozygosity and fitness‐related traits, we fitted separate linear mixed‐effects models for the two fitness‐related traits described above: body mass and horn growth using the package lme4 (Bates, Maechler, Bolker, & Walker, 2015) in R (version 3.2.4: R Core Team, 2016). We then performed model selection following the approach proposed by Grueber, Nakagawa, Laws, and Jamieson (2011) and Burnham, Anderson, and Huyvaert (2011). Based on our hypothesis and on a literature survey, we selected a set of factors to be included in the fixed part of the models for both traits. Multilocus heterozygosity at neutral markers has been shown to explain a variation in fitness‐related traits in Brambilla et al. (2015) but it was not correlated with multilocus heterozygosity at the MHC in this study (see 4). Hence, we included MHC heterozygosity and neutral heterozygosity. We also included age and age^2^ as well as the interactions MHC heterozygosity*age and neutral heterozygosity*age in the set of fixed covariates as both body mass and horn growth are known to vary with age following a quadratic curve (Bergeron, 2007; von Hardenberg, 2005). As we had repeated phenotypic measures for individuals and measures taken in different years, we added individual identity and year as random effects in all models. We then built a set of models (reported in Table 1) based on the hypothesis of this study and we compared them using the Akaike's information criterion (AICc). As we needed to select among models with differing fixed effects, we used maximum‐likelihood estimates. We chose a ΔAICc = 4 for the selection of the set of best models and we obtained standardized coefficients of the variables through natural model averaging among the selected models (REML estimates) as suggested by Grueber et al. (2011). Pseudo‐*R* ^*2*^ (*R* ^*2*^m and *R* ^*2*^c) values were obtained for the best models with the r.squaredGLMM function of the R package {MuMIn} (Barton, 2016). Marginal *R* ^*2*^ (*R* ^*2*^m) represents the variance explained by fixed factors, and conditional *R* ^*2*^ (*R* ^*2*^c) represents the variance explained by both fixed and random factors.

### 3.3 Effect of specific MHC markers and alleles

Fitness‐related traits may be correlated with multilocus heterozygosity (as described above), with heterozygosity at specific markers or with the presence of specific alleles. To investigate the effect of specific MHC markers, we built separate linear mixed‐effects models for each locus for the fitness‐related traits (genetic models). We selected the four MHC‐linked markers (OLADRB1, OLADRB2, OMHC1, and Bf94.1) known to be located within the MHC class I, II, and III. The structure of the models was similar to that described in the previous section. The fixed part of the model included heterozygosity of the selected MHC‐linked marker (H), age, and age^2^. As the interaction between age and the genetic term was never included in the set of the best models of the previous analyses, we did not include the interaction H*age. Individual identity and year were added as random effects in all models. In order to evaluate whether the models explaining fitness‐related traits in function of age improved with the addition of the genetic terms, we also compared the genetic models with models that did not include heterozygosity (age models) choosing a value of ΔAICc = 4 as a threshold. If ΔAICc was <4, standardized coefficients were obtained through natural model averaging between age and genetic models (Burnham et al., 2011).

We also tested for an association between fitness‐related traits and single alleles of OLADRB1, OLADRB2, OMHC1, and Bf94.1. For each locus, the possibility of an association between different alleles and the fitness‐related trait was assessed under an additive model that included the different alleles as genetic terms and scored allele counts as 1 if the allele was present in one copy, 2 if present in two copies, and 0 if it was absent. In each model, we only included the allele counts of *k‐1* alleles to account for the complete dependence of the last allele.

### 3.4 Infectious keratoconjunctivitis

The effect of multilocus heterozygosity at the MHC and of heterozygosity at single loci on individual probability of keratoconjunctivitis infection (IKC) was tested with binomial generalized linear models. The occurrence of the disease during the outbreak was used as a binomial dependent variable and heterozygosity (multilocus or at single loci) was added to the set of explanatory variables (see Table 2 for model specification). To allow for possible age effects, we also added age to the set of explanatory variables. Model building, model selection and model averaging were performed as described for body mass and horn length (see above).

**Table 2:** Description of the fixed part of the binomial generalized linear model set built to test for correlations between heterozygosity and infectious keratoconjunctivitis (IKC)

Model description	Model notation of the fixed effect	AICc
Age	logit (pIKC)_*ij*_ = **β**_***0***_ * + * **β**_***1***_ *·*Age_i*j*_ * + *ε_*ij*_	**86.400**
MLH MHC	logit (pIKC)_*ij*_ = **β**_***0***_ * + * **β**_***1***_ *·*MLH_MHC_*ij*_ * + *ε_*ij*_	**82.695**
Age, MLH MHC	logit (pIKC)_*ij*_ = **β**_***0***_ * + * **β**_***1***_ *·*Age_*ij*_ * + * **β**_***2***_ *·*MLH_MHC_*ij*_ + ε_*ij*_	**84.039**
Age, both MLH	logit (pIKC)_*ij*_ = **β**_***0***_ * + * **β**_***1***_ *·*Age_*ij*_ * + * **β**_***2***_ *·*MLH_n_*ij*_ * + * **β**_***3***_ *·*MLH_MHC_*ij*_ * + *ε_*ij*_	**85.222**

Models with AICc values reported in boldface (ΔAICc < 4 compared to the best model) were used for model averaging.

MLH, multilocus heterozygosity.

## 4 RESULTS

### 4.1 Genetic diversity and heterozygosity in the Gran Paradiso population

A first aim of this study was to estimate the genetic variation at the MHC region in Alpine ibex of the source of all existing populations: the Gran Paradiso population. The diversity measures of the six MHC‐linked microsatellites in the Gran Paradiso population are reported in Table 3. Multilocus heterozygosity at neutral microsatellites was 0.434 ± 0.005 (mean ± standard error), and multilocus heterozygosity at MHC was 0.379 ± 0.014. None of the six MHC‐linked microsatellites was out of Hardy–Weinberg equilibrium. The MHC‐linked microsatellite OLADRB1 was in significant linkage disequilibrium with the three other microsatellites within the MHC region (OLADRB2, OMHC1, and Bf94.1, *p *<* *.001).

**Table 3:** Genetic diversity at six MHC‐linked markers (all samples of Gran Paradiso population, *N* 247)

Locus	Na	*A*_E_	*H*_O_	*H*_E_	*G*_IS_
Bf94.1	3	1.755	0.408	0.431	0.054
BM1258	5	3.827	0.740	0.740	0.001
BM1818	2	1.338	0.272	0.253	−0.074
OLADRB1	3	2.051	0.432	0.514	0.159
OLADRB2	2	1.044	0.034	0.042	0.184
OMHC1	4	1.585	0.349	0.370	0.057
37 neutral loci	3.54	2.02	0.435	0.455	0.045

Na, number of alleles observed; *A*_E_, effective number of alleles (=number of equally frequent alleles it would take to achieve a given level of gene diversity); *H*_O_, observed heterozygosity; *H*_E_, expected heterozygosity; *G*_IS_, deviation from HWE.

Mean number of alleles at MHC‐linked microsatellites was 3.17 (mean *A*_E_: 1.93).

The genetic diversity at the MHC‐linked markers was comparable to what was observed at five of the six markers among 39 populations in Switzerland, which were all reintroduced and indirectly originated from the Gran Paradiso population (Grossen et al., 2014). However, only three OLADRB1 alleles rather than four as in the Swiss populations were found. One of these three microsatellite alleles, OLADRB1 184, has previously been shown to be diagnostic for introgression at the MHC DRB exon II (Grossen et al., 2014). This allele was confirmed to be at very low frequency in GP (2.7%), as also suggested by a previous study performed with a smaller sample size (Grossen et al., 2014). The observation of no previously undetected allele at the microsatellite OLADRB1 suggests no additional introgressed haplotype in Gran Paradiso.

**Figure 1:**
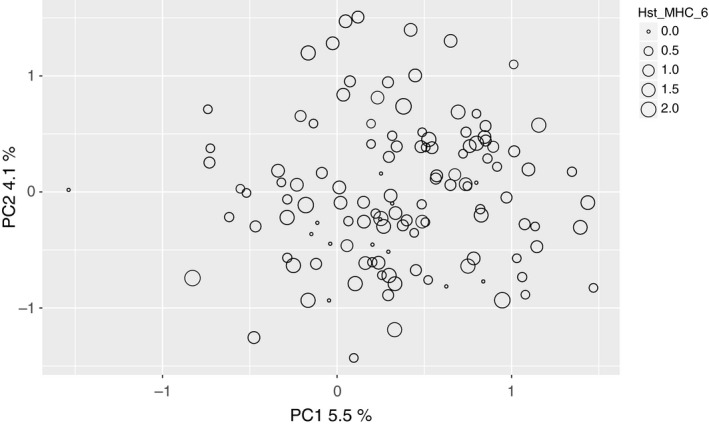
Principle component analysis based on 37 microsatellites of the Gran Paradiso individuals used for the heterozygosity–fitness correlations. Each circle represents an individual, and the size of the circle shows its multilocus heterozygosity at the MHC markers

We were interested in potential heterozygosity–fitness correlations at the MHC. To exclude the possibility that such a correlation simply reflects a genome‐wide heterozygosity–fitness correlation, we compared genome‐wide and MHC heterozygosity. Multilocus heterozygosity at neutral and MHC microsatellites were not correlated (linear regression: β ±* SE* = 0.018 ± 0.022; *p = *.413 n.s.), indicating that heterozygosity at MHC‐linked microsatellites was independent from that at neutral microsatellites. Randomly subsampling six neutral loci produced significant correlations with heterozygosity including all neutral markers (data not shown), suggesting that the lack of correlation of MHC heterozygosity and neutral heterozygosity was not due to low statistical power. Individuals with similar MHC heterozygosity were not more related to each other than individuals with different MHC heterozygosity (no clustering evident in Figure 1).

**Figure 2:**
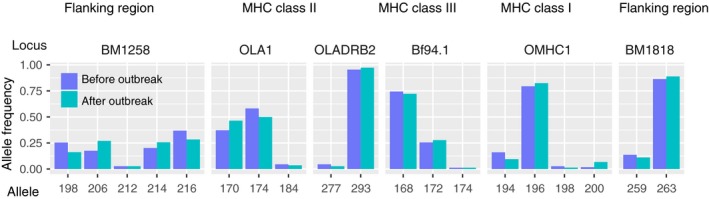
Allele frequencies of all six MHC markers calculated for individuals (*N* = 57) sampled before the disease outbreak (from 2002 to 2004, purple) and individuals (*N* = 37) sampled after the disease outbreak (from 2009 to 2011, green). Figure S1 for allele frequencies among individuals sampled before 2001

Allele frequencies of individuals sampled immediately before and after the disease outbreak are represented in Figure 2 (Figure S1 and Table S1 for allele frequencies of individuals sampled before 2001). In three markers, BM1258, OLADRB1, and BF94.1, the allele frequencies changed toward a more even distribution among alleles from before to after the disease outbreak with smaller variance of allele frequencies found after the outbreak (Figure 2). However, a resampling analysis performed randomly attributing 1,000 times the individuals to either of the two time categories, showed that changes in allele frequency of the same magnitude can simply arise by chance, hence by drift or just due to the sampling (results in Table S2). In the case of marker OLADRB1, for instance, 355 of 1,000 resampling iterations lead to a change in allele frequencies (here difference in allele frequency variance) equal to or smaller than the observed one, corresponding to a probability *p *=* *.355; such an allele frequency change may occur by chance.

### 4.2 Heterozygosity–Fitness correlation

The power to detect heterozygosity–fitness correlations with multiple loci is higher in the presence of identity disequilibrium (here measured as g_2_). The estimate of g_2_ for the MHC‐linked loci was significantly different from 0 (g_2_ ± *SD* = 0.126 ± 0.043, *p* = .001) showing the presence of identity disequilibrium in MHC‐linked microsatellites, and the condition for detecting heterozygosity–fitness correlations was provided.

**Figure 3:**
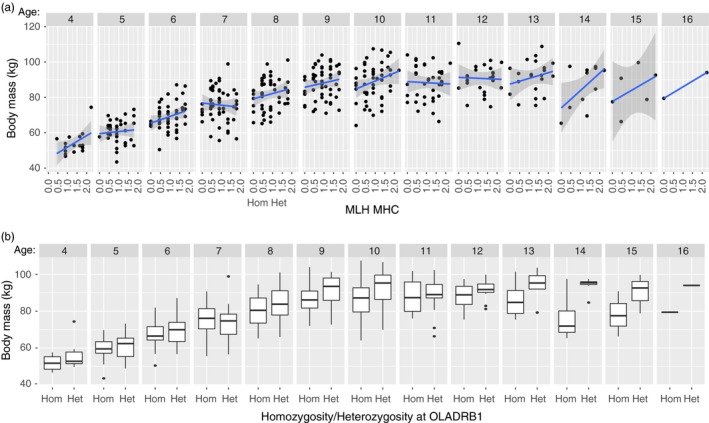
(a) Correlation between body mass and multilocus heterozygosity (MLH) at the MHC shown individually for each age class (from age 4 to age 15); parameter estimates of the corresponding model are presented in Table 4. (b) Boxplots of body mass and heterozygosity of OLADRB1 shown individually for each age class (from age 4 to age 15). Parameter estimates of the corresponding model are presented in Table 5. The number of observations for each age class was as follows (age = *N*): age 4 = 16; age 5 = 37; age 6 = 47; age 7 = 54; age 8 = 50; age 9 = 54; age 10 = 49; age 11 = 38; age 12 = 25; age 13 = 21; age 14 = 12; age 15 = 6

In wild species with long generation times, as is the case in Alpine ibex, it is often necessary to use fitness proxies to investigate heterozygosity–fitness correlations (Brambilla et al., 2015). Using a mixed‐effects model, we tested for the effect of multilocus heterozygosity (MHC and neutral) and age on body mass and horn growth. As expected, both traits varied as a function of age. Neutral heterozygosity had positive but no significant effect on body mass and horn growth (Table 4). However, direction and coefficients of neutral heterozygosity were comparable with those presented in Brambilla et al. (2015). The effect of MHC heterozygosity was significant only for body mass and showed a positive effect (Figure 3a and Table 4).

**Table 4:** Standardized averaged coefficient of the mixed‐effects models built to test heterozygosity–fitness correlations

Parameter	Model Body massβ ± SE, (Confidence intervals)	Model Horn Growth β ± SE, (Confidence intervals)
MLH neutral	0.912 ± 1.483, (−1.995, 3.819)	0.227 ± 0.186, (−0.137, 0.591)
MLH MHC	**3.774 ± 1.642, (0.556, 6.992)**	0.001 ± 0.021, (−0.039, 0.042)
Age	**21.360 ± 0.829, (19.735, 22.985)**	−**1.637 ± 0.126, (**−**1.884,** −**1.390)**
Age^2^	−**21.176 ± 0.973, (**−**23.083,** −**19.269)**	−**1.178 ± 0.174, (**−**1.520,** −**0.837)**
MLH neutral*age	−0.985 ± 1.198, (−3.333, 1.363)	0.014 ± 0.086, (−0.155, 0.183)
MLH MHC*age	−1.303 ± 1.219, (−3.692, 1.086)	0.002 ± 0.026, (−0.049, 0.052)
*R* ^*2*^ m, *R* ^*2*^ c	.601, .909	.290, .409

A separate set of models was built for each of the fitness‐related traits. Coefficients were obtained through natural model averaging between models with ΔAIC < 4 compared to the best model. Values in boldface represent coefficients and confidence intervals that did not overlap zero. The last line of the table reports *R* ^*2*^ m and *R* ^*2*^ c for the best model for each trait. *R* ^*2*^ m and *R* ^*2*^c represent marginal and conditional pseudo‐*R* ^*2*^‐values.

### 4.3 Effect of specific MHC markers and alleles

Correlations between MHC variation and fitness may either be found with multilocus heterozygosity, heterozygosity at specific markers, or the presence of specific alleles. We therefore also tested for the effect of heterozygosity at specific MHC markers on body mass and horn growth (Table 5). The two MHC‐linked microsatellites OLADRB1 and Bf94.1 showed a weak positive effect of heterozygosity on body mass (Table 5 and Figure 3b).

**Table 5:** Summary table of the separate models built for each marker and each trait

Genetic marker	Body mass β ± SE, (Confidence intervals)	Horn growth β ± SE, (Confidence intervals)
OLADRB1	**4.406 ± 1.817, (0.845, 7.967)**	0.006 **±** 0.161, (−0.310, 0.322)
OLADRB2	0.159 ± 4.329, (−8.326, 8.644)	−0.289 ± 0.543, (−1.353, 0.775)
OMHC1	−0.675 ± 1.652, (−3.91, 2.563)	0.061 ± 0.146, (−0.225, 0.347)
Bf94.1	**3.406 ± 1.709, (0.056, 6.756)**	0.022 ± 0.156, (−0.284, 0.328)

For each model, averaged standardized coefficients, SE, and confidence intervals of the genetic term are presented. Model average was performed on genetic and age model when Δ AICc between the two was <4. Values in boldface represent coefficients and confidence intervals that did not overlap zero.

We furthermore built models including additive allele effects to test for effects of specific alleles. All models for body mass and horn growth that tested for additive allele effects performed worse than the models without genetic effect (Δ AIC > 4) and were therefore rejected.

### 4.4 Infectious keratoconjunctivitis

**Figure 4:**
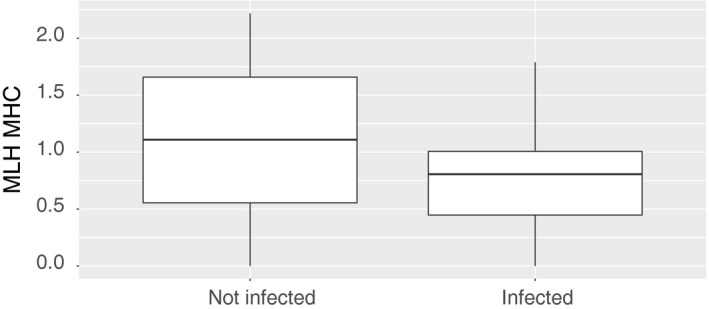
Boxplot showing multilocus heterozygosity at the MHC (MLH MHC) of individuals that did not show infectious keratoconjunctivitis symptoms (not infected, *N* = 41) compared to symptomatic individuals (infected, *N* = 25). Parameter estimates of the corresponding model are presented in Table 6

The population of Gran Paradiso has suffered from an outbreak of infectious keratoconjunctivitis between 2005 and 2008. Of 66 marked individuals monitored during the outbreak, 25 showed symptoms of infectious keratoconjunctivitis. At the end of the outbreak, 20 individuals were observed after recovering. We tested for a correlation between MHC heterozygosity and the risk of showing symptoms of the disease during the outbreak. To test for correlations with genome‐wide heterozygosity, the model also included neutral heterozygosity. MHC heterozygosity showed a negative correlation with infectious keratoconjunctivitis detection (Table 6). Individuals with lower heterozygosity more likely showed infectious keratoconjunctivitis symptoms (Figure 4). Neither neutral heterozygosity nor age was correlated with infectious keratoconjunctivitis. The same result was confirmed using a Cox proportional hazard regression model (data not shown).

**Table 6:** Summary table of the conditional standardized averaged coefficients for the logistic regression built to test for a correlation between infectious keratoconjunctivitis, heterozygosity, and age

Parameter	β ± SE, (Confidence intervals)	Odds ratio, (CI 2.5%–97.5%)
MLH neutral	1.886 ± 2.103, (−2.236, 6.008)	6.600, (0.098, 444.050)
MLH MHC	−**0.915 **±** **0.462, **(**−**1.821,** −**0.009)**	0.401, (0.160, 1.008)
Age	0.065 ± 0.084, (−0.100, 0.230)	1.068, (0.902, 1.263)

Pseudo‐*R* ^*2*^ of the full model = .11. MLH, multilocus heterozygosity. *N* = 66. Values in boldface represent coefficients and confidence intervals that did not overlap zero.

As the observed correlation of MHC heterozygosity may be driven by mainly one marker, we also performed an analysis per locus. Heterozygosity at the microsatellite OLADRB1 showed a significant negative effect on infection probability (β ± *SE* = −1.404 ± 0.654, confidence intervals: {−2.686, −0.123}, Mc Fadden *R* ^*2*^ **= .317), while no relationship was found with heterozygosity at any of the other microsatellites within the MHC region (OLADRB2, OMHC1, and Bf94.1).

Not only heterozygosity at certain loci may be correlated with disease susceptibility but also specific alleles. We found no evidence for additive effects of the presence of specific alleles at the four MHC microsatellites.

## 5 DISCUSSION

The aims of this study were to precisely estimate the genetic variation at the MHC in the source of all existing Alpine ibex populations, the Gran Paradiso one, and to investigate whether individual variation at the MHC was correlated with disease susceptibility or other fitness‐related traits. We found very low genetic variation at the MHC and evidence for heterozygosity–fitness correlations. Individuals with higher heterozygosity at the MHC showed higher body mass and were less disease susceptible during an outbreak of infectious keratoconjunctivitis.

### 5.1 Low genetic diversity at the MHC

The results of this study, conducted on 247 individuals of the population of Gran Paradiso, demonstrated very low genetic variation at the MHC in Alpine ibex. This is in accordance with previous findings based on a considerably smaller sample size (Grossen et al., 2014). The low variability at the MHC region, as well as low genome‐wide variability demonstrated in previous studies (Biebach & Keller, 2009; Brambilla et al., 2015; Grossen et al., 2017), is likely explained by the recent bottleneck experienced by Alpine ibex (Grodinsky & Stuwe, 1987). Although the MHC markers used in this study were originally developed for sheep and cattle, ascertainment bias is unlikely to explain the very low diversity observed. The same MHC microsatellites were successfully used in several related species of the Bovidae family and showed, for instance, 17 alleles in 86 domestic goat individuals (Schwaiger et al., 1993).

### 5.2 Heterozygosity–fitness correlations at MHC markers

We analyzed an outbreak of infectious keratoconjunctivitis, which occurred in the Gran Paradiso area between 2005 and 2008, and we found that individuals with lower heterozygosity at the MHC were more likely to show symptoms of the disease. The pathogenesis of infectious keratoconjunctivitis is thought to be influenced, among other factors, by host predispositions (Mavrot et al., 2012). Male ibex in the study area of Levionaz live in fission–fusion groups and have repeated contact with each other throughout the year except for late winter and early spring. They were thus all likely to have come into contact with the pathogen. However, not all of them showed disease symptoms during the outbreak and we found that individual heterozygosity at the MHC may explain at least some of the variance in disease susceptibility observed among individuals. Although infectious keratoconjunctivitis does not directly lead to death, the blindness caused by the disease often leads to death by starvation or falls. As a consequence, the mortality during an outbreak can reach 30% in some populations (Giacometti et al., 2002). Populations with high MHC heterozygosity may therefore be less prone to severe reductions as a consequence of the disease. During the outbreak of Gran Paradiso, nearly 80% of the diseased marked individuals observed in the Levionaz study site recovered (B. Bassano, unpublished data). Unfortunately, data about the seriousness of the symptoms were not available and the number of individuals known to have died before recovering was not large enough to test whether they had different heterozygosity compared to the ones that recovered after the infection. A large epidemiological study (Gelormini et al., 2017) has identified several strains of *Mycoplasma conjunctivae* in the same and in different populations. The seriousness of symptoms and the disease morbidity of the same strains differed among populations. Although the effect of the environment cannot be excluded, such differences may reflect differences in genetic variation, both at individual and at population level. The latter is consistent with our finding that individuals with different genetic composition show different disease susceptibility.

We showed that MHC heterozygosity may affect disease susceptibility; moreover, we found that heterozygosity at MHC was positively correlated with body mass: Individuals with higher heterozygosity were heavier (Figure 2a). We did not find interactions between MHC heterozygosity and age, indicating that the effect of MHC heterozygosity is visible at all ages. Body mass in Alpine ibex is a trait closely linked to fitness: Heavier males tend to be of higher dominance status (Bergeron, Grignolio, Apollonio, Shipley, & Festa‐Bianchet, 2010), and hence, body mass probably also affects reproductive success (Willisch et al., 2012). Moreover, in ungulates, body mass is correlated with other traits important for fitness, as, for instance, horn growth (Brambilla et al., 2015) and can affect survival, although this effect has been found only for some sex‐age classes (Festa‐Bianchet, Jorgenson, Bérubé, Portier, & Wishart, 1997; Gaillard, Festa‐Bianchet, Delorme, & Jorgenson, 2000; Loison, Langvatn, & Solberg, 1999; Nussey et al., 2011). Finally, body mass may also be associated with pathogen resistance as suggested by Ditchkoff, Lochmiller, Masters, Hoofer, and Van Den Bussche (2001) in a study conducted on white‐tailed deer (*Odocoileus virginianus*).

Due to differing habitat use, females are extremely difficult to mark. Therefore, the long‐term data set is nearly exclusively based on males. We consequently decided to only use males in this study. However, selective pressure might act differently in males and females: Body mass is more crucial for survival and reproductive success of females (Loison et al., 1999), and consequently, heterozygosity–fitness correlations may be different in the two sexes.

We would instead expect similar findings in males and females regarding infectious keratoconjunctivitis. Because of the extremely high morbidity of *Mycoplasma conjunctivae*, both sexes are likely to have the same probability of being in contact with the pathogen. In support of this, no differences in prevalence of infection have been reported between male and female Alpine ibex (Ryser‐Degiorgis et al., 2009).

The MHC region is primarily known to be involved in the immune response (Siddle, Marzec, Cheng, Jones, & Belov, 2010), and thus, higher variation at the MHC region is expected to be favoured. Individuals carrying a range of MHC alleles are more successful in fighting diseases as they produce a larger number of MHC proteins that bind immunogenic peptides for the activation of an immune response (Janeway et al., 2001).

Evidence for correlations between MHC variation and survival or disease susceptibility was also found in other wild populations, some of which are endangered species. This indicates that MHC variation is likely to play an important role in species conservation. Paterson et al. (1998) found that allelic variation at the MHC was associated with juvenile survival and resistance to intestinal nematodes in Soay sheep (*Ovis aries*). Specific alleles of the MHC were also found to be related to survival in the endangered Attwater's prairie‐chicken (*Tympanuchus cupido attwateri*, Bateson et al., 2016). A study by Siddle et al. (2007) provided a direct link between susceptibility to contagious cancer and low MHC class I diversity in Tasmanian devil (*Sarcophilus harrisii*). More recently, Osborne et al. (2015) found heterozygote advantage at DRB locus in response to bacterial infection in the threatened New Zealand sea lion (*Phocarctos hookeri*), and Aguilar et al. (2016) found an association between specific MHC class I alleles and infection intensity of a blood parasite in the blue tit (*Cyanistes caeruleu*s).

Our results suggest an advantage for individuals with higher heterozygosity. Heterozygote advantage is expected to lead to deviations from Hardy–Weinberg equilibrium. However, none of the six MHC‐linked microsatellites analyzed in this study was out of Hardy–Weinberg equilibrium, suggesting that neutrality cannot be excluded. Note, however, that for this test, lack of power may be an issue.

As expected under heterozygote advantage, the allele frequencies of the marker OLADRB1 (which showed a significant per locus correlation with IKC susceptibility) but also of two other MHC‐linked markers changed toward a more even allele frequency distribution from before to after the disease outbreak. Although this is consistent with heterozygote advantage at OLADRB1 (see also below), a resampling analysis showed that this outcome may simply be explained by chance effects (drift and/or sampling).

### 5.3 Direct effects of MHC markers versus general genome‐wide effects

Heterozygosity–fitness correlations have previously been reported in the same population for neutral markers (Brambilla et al., 2015). Heterozygosity–fitness correlations at neutral microsatellites are a signal of inbreeding depression and can be considered as evidence of the “general effect” hypothesis that states that heterozygosity at neutral microsatellites is representative of genome‐wide heterozygosity, which is advantageous for individuals (Chapman, Nakagawa, Coltman, Slate, & Sheldon, 2009; Hansson & Westerberg, 2002). MHC‐linked microsatellites, instead, are closely linked to genes under selection (Paterson, 1998; Paterson et al., 1998); a correlation between heterozygosity and fitness can thus be an evidence of the “direct effect”: the advantage of heterozygosity at loci directly affecting fitness (Hansson & Westerberg, 2002). Identity disequilibrium, a correlation between heterozygosity and homozygosity across loci, was found among the MHC markers providing the conditions for heterozygosity–fitness correlations to arise (Szulkin, Bierne, & David, 2010).

We performed several tests in order to disentangle general from direct effects. Multilocus heterozygosity at neutral markers and at MHC markers was not correlated, and no clustering of individuals with similar multilocus heterozygosity at the MHC was found in a principal component analysis based on neutral markers. Furthermore, we included both neutral and MHC heterozygosity in our models. For body mass, we found evidence for heterozygosity–fitness correlations both with neutral and with MHC heterozygosity. Accounting for different standardization methods, direction and coefficients of neutral heterozygosity were comparable with those presented in Brambilla et al. (2015) (neutral multilocus heterozygosity was positively correlated with body mass and horn growth and negatively correlated with the number of intestinal parasite eggs). The model including MHC heterozygosity explained more variation than if only including neutral heterozygosity. In the case of infectious keratoconjunctivitis, only MHC heterozygosity had an effect on the probability of infection, while no relationship with neutral heterozygosity was found. All these findings suggest that the two sets of markers were statistically independent and that the correlations found with MHC heterozygosity were not simply genome‐wide effects but direct effects of the MHC on the analyzed traits.

Several studies on heterozygosity–fitness correlations as well as the previous findings on this species (Brambilla et al., 2015) and the results of this study demonstrated that both genome‐wide and MHC heterozygosity play a role in fitness. Yet, we have also found that genome‐wide heterozygosity and heterozygosity at MHC are not necessarily correlated. As we explain in the conclusions, this finding may have important consequences on management strategies based on the selection of high genetic variation but, to our knowledge, such correlations are only rarely looked at.

The markers used in this study to calculate MHC heterozygosity were in strong linkage and identity disequilibrium with each other. It was therefore not possible to pinpoint the region responsible for the effects found on fitness. However, other studies found heterozygosity–fitness correlations with single MHC genes rather than multilocus heterozygosity. To explore the possibility that the heterozygosity–fitness correlation at the MHC was mainly driven by one of the markers, we also tested for correlations at each locus. We indeed found that heterozygosity of OLADRB1 was positively correlated with body mass and resistance to infectious keratoconjunctivitis. Hence, the heterozygote advantage observed at the MHC may be related to heterozygote advantage at the marker OLADRB1, which in turn might be explained by direct selection potentially acting on MHC genes closely linked to that microsatellite as suggested by Da Silva et al. (2008). The microsatellite OLADRB1 is adjacent and closely linked to the second exon of DRB (Schwaiger et al., 1993). This exon encodes an antigen‐binding site and is known for generally extremely high polymorphisms (for instance, several hundred alleles in humans). In Alpine ibex, four alleles were found at the microsatellite OLADRB1, but only two different exon sequences (Alasaad et al., 2012; Grossen et al., 2014). One of the four microsatellite alleles (184) was found to be diagnostic for one of the two exon sequences (Caib‐DRB*2, Alasaad et al., 2012; Grossen et al., 2014). Caib‐DRB*2 was also shown to have introgressed from domestic goat (Grossen et al., 2014). All three remaining microsatellite alleles occurred with the same other Alpine ibex DRB exon II allele (Caib‐DRB*1). We confirmed the findings by Grossen et al. (2014) that allele 184 was rare in the population of Gran Paradiso (2.7%). Therefore, the correlations that we found with heterozygosity at OLADRB1 were mainly due to genotypes not involving allele 184 and may hence mostly involve homozygotes for the sequence allele Caib‐DRB*1. The observation that the allele frequency of 184 (and the completely linked allele 277 of marker OLADRB2) barely changed from before to after the outbreak, while the frequencies of alleles 170 and 174 changed to nearly equally frequent, is consistent with this interpretation. We therefore expected that functional heterozygosity close by, but not at the second exon of DRB, may be related to the observed differences in body mass and disease susceptibility. The observation of decreased disease susceptibility for individuals, which were heterozygous in the region of DRB, shows that variation in this gene region is meaningful and supports the hypothesis that the introgression was adaptive (Grossen et al., 2014).

While we found higher fitness for heterozygotes, we did not find an effect of specific MHC alleles neither on life history traits nor on susceptibility to infectious keratoconjunctivitis. An effect of specific alleles on infection susceptibility was instead found in many studies as, for example, in frogs (Savage & Zamudio, 2011), in the Chinese egret (Lei et al., 2016), and in blue tits (Aguilar et al., 2016). Our results would therefore be in accordance with heterozygote advantage rather than the effect of specific alleles. However, also a lack of statistical power can explain these findings.

### 5.4 Management implications

After a severe bottleneck at the beginning of 19th century, Alpine ibex recovered and, thanks to a very successful reintroduction program, are now again spread across the entire Alpine arc (around 50,000 individuals counted on the Alps in 2013, GPNP, unpublished data). However, genetic consequences of the reintroduction history, including low genetic diversity and strong population structure, are still visible (Biebach & Keller, 2009; Grossen et al., 2017).

During the last years, direct and indirect consequences of epidemic diseases affected several, both recently introduced and established, populations. Examples are sarcoptic mange in the Eastern Alps (Carmignola et al., 2006), brucellosis in Bargy massif (Mick et al., 2014), respiratory diseases in Vanoise (Garnier et al., 2016), and infectious keratoconjunctivitis in several populations, including the source of all existing Alpine ibex populations, the Gran Paradiso population. Our study highlighted that low genetic variation at the MHC region can affect body mass, a trait related to fitness, and provided first evidence that variation at the MHC may affect resistance to a certain pathogen, the *Mycoplasma conjunctivae* in this species. Although our study was performed only in one population, the genetic diversity at the MHC found in Gran Paradiso is similar to what was found in other populations (Grossen et al., 2014), indicating that the concern and potential application presented in this study are valid for the entire species.

Our results confirmed that low variation at the MHC can become an issue in the case of disease outbreaks, but we were not able to identify specific genes involved in the process demonstrating that the dynamic between genetic variation and disease resistance (more in general, also fitness) is complex and caution has to be taken when applying these results to management and conservation strategy. Indeed, we found that heterozygosity at the MHC is not necessarily related to genome‐wide heterozygosity but the latter has previously also been shown to be correlated with fitness (Brambilla et al., 2015). Although it may be tempting to plan reintroduction and restocking actions selecting individuals for their heterozygosity at specific gene regions (e.g., genes involved in the immune response), this has to be avoided as it may lead to the selection of individuals with lower genome‐wide variation or lower variation at other less investigated regions. A careful solution for planning translocation might be to choose unrelated individuals (based on genome‐wide marker information), and from these, prefer individuals with maximal heterozygosity at the MHC or choose individuals with rare alleles to maximize allelic diversity in the target population. To take genetic variation into account deciding management strategies is still not common practice (Frankham, Ballou, & Briscoe, 2002). If genetics is considered, it is currently still mainly only based on neutral variation, as, for instance, a recent study on Galapagos tortoises shows (Miller et al., 2017).

In light of our results and the most recent findings on infectious keratoconjunctivitis (Gelormini et al., 2017), some considerations arise also on management strategies in the case of an outbreak. A strategy sometimes used during infectious keratoconjunctivitis outbreaks in managed populations is the withdrawal of individuals as soon as they show symptoms in order to reduce the spread of the disease. This strategy may appear optimal as it is expected to preferentially remove individuals with lower genetic quality at immune‐related loci (here lower heterozygosity at the MHC). However, our results show that it may also remove genome‐wide variation from the population that is important for individual fitness (Brambilla et al., 2015) and for population performance (C. Bozzuto, personal communication). Together with evidence from disease dynamics, it is questionable whether removal of diseased animals is a good strategy.

Finally, in a conservation framework, it became crucial to increase the knowledge on the relationship between variation at disease‐relevant loci as, for instance, the MHC and disease susceptibility in wild bottlenecked populations in order to inform management decisions.

## CONFLICT OF INTEREST

We confirm there is no actual or potential conflict of interest including any financial, personal, or other relationships with people or organizations that could inappropriately influence our work. .
