# Supplementary material for: Heterozygosity–fitness correlation at the major histocompatibility complex despite low variation in Alpine ibex (Capra ibex)
Source: Evol Appl. 2017 Dec 4;11(5):631–44. doi: 10.1111/eva.12575 (PMC5979623; doi:10.1111/eva.12575)
Supplement: Supplementary file 1 [file EVA-11-631-s001.pdf]

Table S1: Allele frequencies at MHC-linked microsatellites estimated for three different sampling times: before 2000, 2001-2004 (before the IKC outbreak), 2009-2011 (after the outbreak)

		Before 2000		Before outbreak		After outbreak	
**Locus**	**Allele**	**Count**	**Frequency**	**Count**	**Frequency**	**Count**	**Frequency**
**BM1258**	198	46	0.27	29	0.25	12	0.16
**BM1258**	206	17	0.10	20	0.18	20	0.27
**BM1258**	212	4	0.02	2	0.03	2	0.03
**BM1258**	214	45	0.26	23	0.20	19	0.26
**BM1258**	216	60	0.35	42	0.37	21	0.28
**BM1818**	259	31	0.18	15	0.14	8	0.11
**BM1818**	263	141	0.82	95	0.86	64	0.89
**Bf94.1**	168	111	0.69	67	0.74	52	0.72
**Bf94.1**	172	47	0.29	23	0.26	20	0.28
**Bf94.1**	174	2	0.01	2	0.01	2	0.01
**OLA1**	170	61	0.41	41	0.37	26	0.46
**OLA1**	174	89	0.59	64	0.58	28	0.50
**OLA1**	184	0	0.00	5	0.05	2	0.04
**OLADRB2**	277	0	0.00	5	0.05	2	0.03
**OLADRB2**	293	162	1.00	105	0.95	72	0.97
**OMHC1**	194	26	0.16	18	0.16	7	0.09
**OMHC1**	196	122	0.76	89	0.79	61	0.82
**OMHC1**	198	5	0.03	3	0.03	1	0.01
**OMHC1**	200	7	0.04	2	0.02	5	0.07

Table S2:

Summary of allele frequency resampling analysis.

Shown are the probabilities that the observed change in allele frequency (here change in allele frequency variance) happened by chance.

Individuals were randomly attributed 1000 times to either of the two time categories (before or after disease outbreak).

For instance marker OLADRB1: in 355 out of 1000 resampling iterations a change in allele frequencies equal or smaller than the observed one was obtained.

Locus	Probability
**BM1258**	0.091
**OLA1**	0.355
**OLADRB2**	0.828
**Bf94.1**	0.398
**OMHC1**	0.659
**BM1818**	0.742
