# Supplementary figures and images for: Heterozygosity–fitness correlation at the major histocompatibility complex despite low variation in Alpine ibex (Capra ibex)
Source: Evol Appl. 2017 Dec 4;11(5):631–44. doi: 10.1111/eva.12575 (PMC5979623; doi:10.1111/eva.12575)

Flanking region

MHC class II

MHC class III

MHC class I

Flanking region

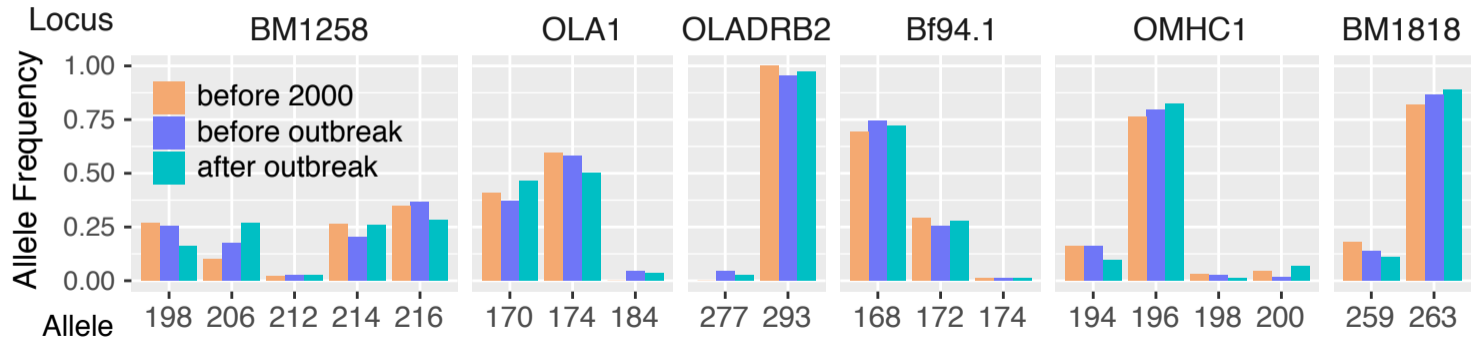

Supplement: Supplementary file 3 [file EVA-11-631-s003.pdf]
