# Supplementary material for: Heterozygosity–fitness correlation at the major histocompatibility complex despite low variation in Alpine ibex (Capra ibex)
Source: Evol Appl. 2017 Dec 4;11(5):631–44. doi: 10.1111/eva.12575 (PMC5979623; doi:10.1111/eva.12575)
Supplement: Supplementary file 4 [file EVA-11-631-s004.docx]

Figure S1:

Allele frequencies of all six MHC markers calculated for Alpine ibex individuals sampled before 2001 (N=87), between 2002 and 2004 (before the disease outbreak, N=57) and between 2009 and 2011 (after the end of the disease outbreak, N=37).
